# Homœopathic Treatment in Cholera

**Published:** 1856-04

**Authors:** 


*Homœopathic Treatment in Cholera.*—The homœopaths have long boasted of their success in the treatment of cholera. The published statistics of their hospitals in Germany and other countries exhibited a rate of mortality highly flattering to the efficacy of "infinitesimals," and well calculated to attract the attention not only of the public generally, but also of the public authorities. Fortunately for the cause of truth and honesty, and we may add, humanity, the authorities of Marseilles, in the south of France, determined, during the late severe epidemic of cholera in that city, to test fairly and openly the truth of what was so boldly asserted. With this object they placed a ward of the Hotel Dieu at the disposal of the leading homœopathic practitioner of the place; and were doubtless prepared to entrust him with the entire management of the hospital, should the peculiar treatment by inconceivable quantities of remedial substances prove more successful than that adopted by the regular practitioner. Dr. Bouquet, writing to the "Gazette des Hopitaux," says — "Homœopathy has just received a severe check in our town. You have perhaps heard of the noise it made last year with its pretended success in cholera. Dr. Chargé asserted that he had not lost one out of several hundred patients, and he published this statement in the political journals of Lyons and Bordeaux. When, during the present year, the scourge visited us anew, the authorities bestirred themselves, and thinking it was their duty to bring the truth to light, they entrusted one of the wards in the Hotel Dieu to Dr. Chargé. There assisted by his colleagues in homœopathy, by pharmaciens, and by some young people his adepts, who devoted themselves to tending the patients, (for he had found the ordinary staff insufficient and incompetent,) he obtained the result which might easily have been anticipated; the broad day-light did not display success. *Of* 26 *cholera patients admitted into this ward,* 20 *died, and M. Chargé withdrew.* To render the experiment conclusive, a ward had been set apart, in which the patients had been treated by rational means, which did not profess to work wonders. *During the same period, of* 25 *patients admitted but* 11 *died. Each ward had its turn of reception.* I think that these facts are sufficiently decisive to render a renewal of such experiments needless, for if science profits by them, which is doubtful, humanity suffers not a little."

We confess to a strong feeling of affection for our kind. Human suffering ever commands our warmest sympathies. We grieve to see an intractable disease seize the strong and the lovely, and, in spite of medical art, hurry them to that country from whose bourne there is no returning. Often have we wished, during the course of the two epidemics of cholera through which we have passed, that some remedy or course of treatment could be discovered whereby the ravages of this fell disease might be stayed. When, therefore, the homœopaths exultingly proclaimed their extraordinary success, we certainly desired that their treatment might be subjected to a fair, impartial trial. This it has now received, and its signal failure will tend to throw discredit on all statements hereafter made by the globulists as to their success in the treatment of epidemic diseases.—*Montreal Med. Chronicle.*

